# The Association of an Electronic Frailty Index with Glaucoma Shunt Outcomes

**DOI:** 10.3390/geriatrics11050114

**Published:** 2026-08-31

**Authors:** Neel Edupuganti, Richa Saxena, Atalie C. Thompson

**Affiliations:** 1 Department of Ophthalmology, Wake Forest University School of Medicine, Winston-Salem, NC 27157, USA; richa.saxena@wfusm.edu (R.S.); atalie.thompson@advocatehealth.org (A.C.T.); 2 MedStar Georgetown/Washington Hospital Center Department of Ophthalmology, Washington, DC 20010, USA

**Keywords:** glaucoma, electronic frailty index, frailty, shunt, drainage device

## Abstract

**Background/Objectives**: Recent studies have shown frailty to be a significant predictor of postoperative complications and mortality across multiple surgical fields. Older adults with glaucoma refractory to medical management often require drainage implantation to achieve intraocular pressure control, yet the impact of frailty remains unstudied. This study evaluated the association of frailty status with glaucoma shunt success and postoperative complications. **Methods**: A retrospective single-center review was performed for older adults who underwent glaucoma drainage device implantation from January 2015 to July 2025. Included patients had a documented electronic frailty index (eFI) score and were followed for up to 1 year of follow-up. Partial surgical success was defined as an IOP of 5–21 mmHg with a ≥20% IOP reduction from baseline at 1 year; complete success additionally required absence of severe complications. Logistic regression assessed predictors of surgical success. Kaplan–Meier and Cox proportional hazards models evaluated time to postoperative complications. **Results**: Among 200 patients/eyes (mean age = 70.6 years), 60% were pre-frail or frail. At 1 year, 79% achieved partial success, and nearly two-thirds had no postoperative complications. Frail/pre-frail status was significantly associated with a higher risk of postoperative complications (HR = 1.90, 95% CI (1.07, 3.36), *p* = 0.027) though not with achieving partial success (OR = 1.49, 95% CI (0.72, 3.07)). There were no significant sociodemographic predictors of postoperative complication timing (all *p* > 0.05). **Conclusions**: Being pre-frail or frail meaningfully increased vulnerability to postoperative complications but was not associated with achieving partial or complete surgical success. The elevated complication risk highlights the importance of frailty assessment in preoperative planning for older adults. Future studies should evaluate how integrating frailty screening into perioperative discussions may optimize outcomes and support shared decision-making in aging patients undergoing glaucoma surgery.

## 1. Introduction

Glaucoma is a leading cause of irreversible blindness and continues to pose a major public health risk [1,2,3]. Glaucoma is characterized by progressive damage to the optic nerve that can lead to visual field loss and eventual vision impairment if not properly controlled. Prevalence studies suggest that primary open-angle glaucoma (POAG) will increase by 50% worldwide, from 52.7 million in 2020 to 79.8 million in 2040, as the population ages [4]. Lowering intraocular pressure (IOP) remains the only proven strategy to slow disease progression [2]. While many patients achieve adequate IOP control through topical medications or laser therapy, a significant number eventually require surgical intervention when conservative measures fail. Among the available surgical options, glaucoma drainage devices (GDDs) have become an important treatment for patients with refractory glaucoma [5].

The most commonly used GDDs are the Ahmed (New World Medical, Rancho Cucamonga, CA, USA) and Baerveldt (Johnson & Johnson Vision, Irvine, CA, USA) implants, which differ in design, surgical technique, and flow mechanism [6]. Multiple comparative studies, including the Ahmed Versus Baerveldt (AVB) study by Christakis et al., have shown that both devices effectively reduce IOP and medication burden in refractory glaucoma at 5-year follow-up [7]. Other studies have reported similar long-term IOP measurements and survival rates between the two devices beyond one year, with variability in postoperative complications and medication reduction [7,8]. Additional work highlights that while both implants are safe and effective [9,10], patient-specific biological responses such as capsular scarring can significantly influence outcomes [11].

Despite extensive comparisons of Ahmed and Baerveldt devices, the impact of frailty on surgical outcomes remains unclear. Prior research prioritized technical metrics over physiologic vulnerability [12], yet frailty is increasingly recognized as a vital predictor of success across many medical and surgical fields. As glaucoma primarily affects older adults, frailty may be an especially relevant factor in how patients tolerate surgery. Frailty is associated with higher rates of postoperative complications, mortality, and impaired functional recovery across other surgical specialties, such as general and vascular surgery [13]. Electronic indices for frailty have emerged as useful scalable tools for quantifying frailty and predicting adverse surgical outcomes [14,15]. However, it is not known whether frailty influences outcomes after glaucoma drainage device surgery. Given the increasing prevalence of both glaucoma and frailty in aging populations [16,17], understanding this relationship is essential to optimize surgical decision-making and postoperative care.

The electronic frailty index (eFI) is a validated, automated tool that compiles 54 clinical deficits from electronic health records using a 2-year lookback window, with weekly score updates for patients aged 55 years or older [18,19]. This cumulative deficit framework is supported by the foundational literature and leverages routine clinical data to predict adverse health utilization, postoperative complications, and mortality [14,15,20,21,22,23].

The goal of this study was to investigate the impact of frailty, as measured by an electronic frailty index (eFI) [20], on surgical outcomes in patients with glaucoma who required implantation of either an Ahmed or Baerveldt glaucoma drainage device. We hypothesized that patients with higher frailty scores would have an increased likelihood of experiencing surgical failure, such as postoperative complications or uncontrolled IOP, compared to less frail patients after one year. By exploring the role of frailty in this surgical population, this study aimed to improve understanding of how systemic health factors influence glaucoma surgery outcomes among older adults, supporting more individualized and risk-informed patient care.

## 2. Materials and Methods

### 2.1. Study Design and Patient Population

A retrospective chart review was conducted for patients at the Atrium Health Wake Forest Baptist Eye Center who underwent Ahmed or Baerveldt glaucoma drainage device implantation from January 2015 to July 2025. Selected patients either had an ICD-9 or ICD-10 glaucoma diagnosis code, a documented electronic frailty index (eFI), and were followed for up to 1 year of follow-up after shunt implantation. Exclusion criteria included age under 18 years, absence of a formal ICD-9 or ICD-10 glaucoma diagnosis, no record of a glaucoma drainage device procedure, evaluation at a site other than the Atrium Health Wake Forest Baptist Eye Center, absence of a calculated eFI within the electronic health record, or clinical encounter dates outside the study period of January 2015 to July 2025. Perioperative care and longitudinal follow-up were both managed by the primary ophthalmology team at the Atrium Health Wake Forest Baptist Eye Center. To account for potential survivor and inclusion bias, all eligible patients were retained in our analyses and censored at the time they were lost to follow-up. This study adhered to the Declaration of Helsinki and received formal approval from the Institutional Review Board (IRB) at Atrium Health Wake Forest Baptist Hospital.

**Figure 1:**
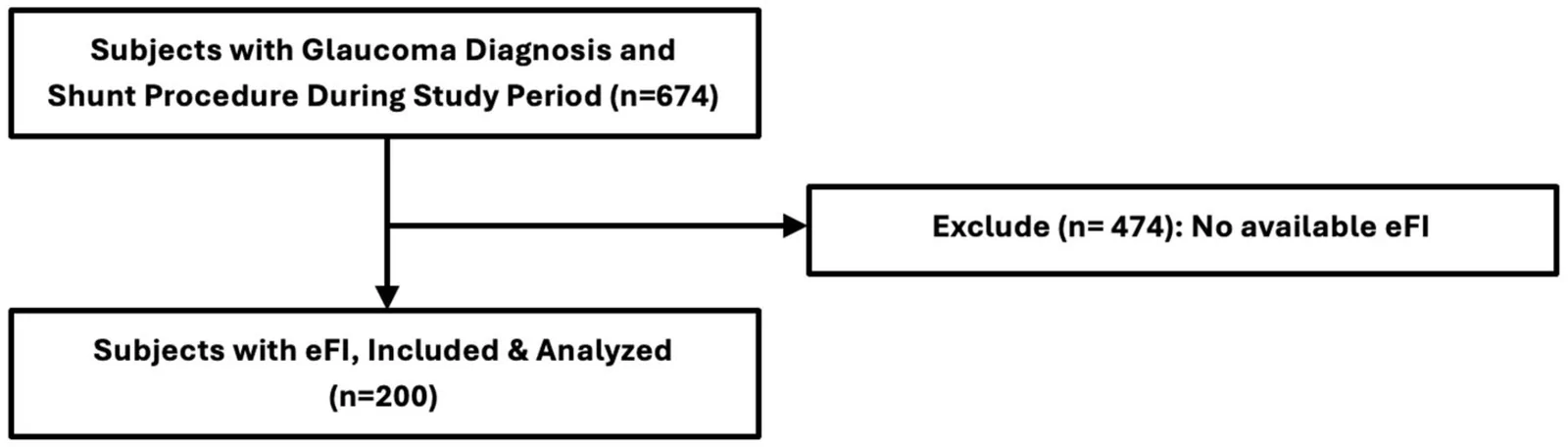
Flowchart of patient cohort selection. Illustration of the funneling process from the initial data warehouse query to the final analytical cohort.

Data extraction was performed using a hybrid approach of automated query and manual chart review. Initial cohort identification was conducted via the i2b2 (Informatics for Integrating Biology and the Bedside) platform, a validated query tool used to extract aggregate data from the Translational Data Warehouse based on the previously mentioned inclusion and exclusion criteria [24]. Subsequent manual abstraction of granular clinical data was performed by a single investigator. The investigator was trained by the senior author on the use of a standardized data collection protocol to ensure consistency. Given this specific data collection process, an inter-rater reliability assessment was not performed. A complete case analysis approach was utilized for this study. Incomplete records were defined as those missing the electronic frailty index. This study was conducted in accordance with the STROBE reporting guidelines and the RECORD statement. A flowchart of the selection process is shown in Figure 1.

### 2.2. Data Collection and Outcome Definitions

Data collected included age, race, ethnicity, marital status, smoking history, surgical history, shunt surgery date, surgery eye, glaucoma diagnosis, and if cataract surgery was performed in combination. Additional collected data included pre-surgical and postoperative follow-up (1 day, 2 weeks, 1 month, 3 months, 6 months, 1 year) intraocular pressure (IOP) and visual acuity (VA). Baseline IOP was defined as the intraocular pressure recorded at the most recent preoperative clinical encounter prior to surgery during which the decision for surgery was made. Other surgical data gathered included medication use and postoperative complications. Severe postoperative complications included tube revision, tube exposure, tube removal, tube infection, endophthalmitis, cyclophotocoagulation (CPC) use, retinal detachment, persistent choroidal effusion/detachment, anterior chamber hemorrhage, persistent hypotony, inflammation, and any cystoid macular edema. Non-severe postoperative complications were those that were temporary or resolved. These postoperative complications were identified via manual abstraction of clinical encounter notes from all follow-up visits with an ophthalmologist and were based on a priori standardized clinical definitions. A comprehensive table of how each postoperative complication was defined and measured throughout the study can be seen in Supplemental Table S1. To minimize detection bias of postoperative complications, the entire clinical record for the first postoperative year was reviewed to ensure all major events were captured.

Surgical success at one year was categorized into two tiers based on IOP control and clinical stability. Partial success was defined as successful IOP reduction (IOP of 5–21 mmHg and ≥20% reduction from preoperative IOP) with resolved non-severe postoperative complications at 1-year postop. Complete success was defined as successful IOP reduction (IOP of 5–21 mmHg and ≥20% reduction from preoperative IOP) at 1-year postop and no postoperative complications. Failure was defined as the occurrence of severe postoperative complications or IOP not being within the above target levels. The number of days after surgery until first postoperative complication was also recorded.

### 2.3. Frailty Categorization and Assessment

We utilized a locally implemented eFI that leverages routine EHR data to identify frailty based on 54 clinical deficits, including diagnoses, vitals, medications, and laboratory values [18,19]. This validated tool predicts acute care utilization and mortality [14,15,18,21,22,23], and it was calculated for patients with at least two outpatient encounters involving blood pressure measurements during a two-year period following the methodology described by Pajewski et al. and updated by Khanna et al. [14,18,19]. Specific variables utilized in the eFI calculation include morbidity characteristics (heart disease, hypertension, liver disease, cancer, etc.), functionality measures (falls, activity limitation, arthritis, peripheral vascular disease, etc.), laboratory measures (body mass index, glucose, cholesterol, kidney function, calcium, etc.), cognitive characteristics (delirium, depression, stress, etc.), and sensory loss (hearing and visual impairment), in addition to other characteristics as outlined in Supplementary Table S2, provided by Khanna et al. [14].

As defined by the eFI, frailty was classified into three categories: fit (eFI ≤ 0.10), pre-frail (0.10 < eFI ≤ 0.21), and frail (eFI > 0.21) [18,19,25]. The eFI was first evaluated as a continuous variable, then as a categorical variable. Due to the small number of subjects in the frail category (*n* = 29), the three-level variable model was underpowered to detect significant associations. Consequently, these categories were further turned into a binary variable (pre-frail/frail vs. fit) for analyses to maximize statistical power and maintain clinical relevance.

### 2.4. Statistical Analysis

Separate univariate and multivariate logistic regression models were used to evaluate factors influencing either partial or complete surgical success. Kaplan–Meier survival curves and Cox proportional hazards regression tests were conducted to evaluate significant factors influencing time to postoperative complication. In the models with eFI, we adjusted for a limited number of a priori demographic variables (age, sex, race, marital status), because the eFI was calculated from multiple other clinical variables [19]. Adjusting for these demographic variables is in keeping with how analyses using the eFI have been done previously [14]. Clinical and surgical covariates (glaucoma subtype, baseline IOP, device type, cataract extraction, prior intraocular surgical history) were also evaluated in bivariate models but were not significant (all *p* ≥ 0.15) and were similarly not significant in multivariable analyses (all *p* > 0.20), so were not retained in the final multivariable model to prevent overfitting [26]. Interaction analysis for frailty x device type was also performed to assess for potential effect modification. All statistical analyses were conducted with JMP Pro 18 (Version 18.2.2). A *p*-value < 0.05 was considered statistically significant.

## 3. Results

### 3.1. Patient Demographics

A total of 200 patients (*n* = 200 eyes) satisfied the inclusion criteria to be included in the study. The mean age of the subjects was 70.6 years (standard deviation (SD) = 9.6 years), with 55% identifying as male and 39% as non-White (Table 1). Most subjects (93.5%) identified as non-Hispanic. Current or previous smokers consisted of 56.5% of the patients. Finally, 80 subjects had an eFI categorizing them as ‘fit’, while the remaining 120 participants were identified as ‘frail’ or ‘pre-frail’.

### 3.2. Clinical Characteristics and Surgical Profiles

Most patients were diagnosed with primary open-angle glaucoma and had severe-stage disease (Table 2). The Ahmed shunt was utilized in 56% of surgeries, with most not being combined with phacoemulsification. More specifically, Ahmed shunts were utilized in 60% of the pre-frail/frail group and 50% of the fit group. There was no significant interaction between frailty status and device type, and device type was not significant in bivariate or multivariate analyses. At 1-year follow-up, 79% achieved successful IOP reduction, while 52% achieved successful IOP reduction with no postoperative complications. While the most common postoperative complication was anterior chamber hemorrhage, nearly two-thirds of patients had no postoperative complications.

### 3.3. Postoperative Complication Analysis

In univariate Cox proportional hazards analysis, frailty status was not associated with time to complication (*p* = 0.118) (Table 3). However, it was significantly independently associated with increased risk of postoperative complications in the multivariable Cox model (HR = 1.90, 95% CI (1.07, 3.36), *p* = 0.027). The Kaplan–Meier survival curve can be seen in Supplementary Figure S1. No demographic variables were significantly associated with decreased risk of postoperative complications. Also, continuous eFI was not associated with the outcomes.

### 3.4. Logistic Regression Analysis of Surgical Success Predictors

In separate logistic regression analyses evaluating either complete or partial success outcomes (Table 4), pre-frail/frail subjects were more likely to achieve partial or complete success, but this was not statistically significant. No significant associations were observed between frailty or sociodemographic predictors and successful shunt procedure outcome.

## 4. Discussion

This study demonstrates that frailty status is associated with an increased risk of post-surgical complications following glaucoma shunt implantation but does not independently predict IOP surgical failure. These findings align with evidence from other surgical fields, where frailty has consistently been identified as a strong determinant of postoperative complications and overall morbidity [27]. In the context of glaucoma care, recent studies using the eFI has shown that frail patients are less likely to receive surgical treatment and often face longer times to surgery [12,28], suggesting that frailty influences both treatment selection and perioperative risk.

Our findings align with growing evidence that frailty serves as a meaningful marker for surgical vulnerability. In ophthalmology, frail individuals have been shown to experience higher rates of adverse events after procedures, such as cataract extraction and vitreoretinal surgery [29], likely due to reduced physiologic reserve, impaired tissue repair, and higher levels of baseline inflammation. The multivariable analysis suggests that frailty exerts an independent effect when demographic and clinical variables are controlled. This pattern is consistent with findings in other surgical fields, where frailty predicts complications more robustly than age or comorbidity burden alone [30,31,32]. These findings highlight that frailty does play a meaningful role in how patients tolerate glaucoma drainage device surgery, even if it does not directly affect IOP outcomes. Although ocular clinical factors, such as glaucoma subtype, device type, and concurrent cataract extraction, could be important facets in pre- and post-surgical planning, they were not significantly associated with the outcomes in this study. This could be a limitation of the cohort selection since the sample size was modest and patients were only included if they had a calculable eFI. Nevertheless, our study findings suggest that in patients receiving care in our health system, a patient’s overall frailty may play a role in the likelihood of early postoperative complications. Therefore, incorporating routine frailty assessments into preoperative planning for GDDs may identify patients who can benefit from closer surveillance and enhanced perioperative support [33]. Since the eFI can be incorporated into EHR clinic notes via an automated dot phrase, it offers a potential means of assessing frailty in glaucoma patients at the point of care. Future larger cohort studies could evaluate whether utilization of such data in surgical decision-making assists surgeons and patients in discussing perioperative risk.

Frailty and demographic factors were not associated with surgical failure as defined by IOP outcomes at 1 year. These findings suggest that the mechanical aspects of the shunt function may be relatively preserved regardless of the patient’s frailty status. This could indicate that IOP reduction after GDD surgery is primarily driven by biomechanical factors, such as plate size, aqueous outflow, or capsular characteristics. Prior studies have shown that the major drivers of shunt success or failure include impaired wound healing, excessive capsular fibrosis, device positioning, and preoperative IOP [34,35]. It is possible that frailty primarily affects recovery processes that lead to complications, rather than the aqueous outflow pathway itself. Other research also emphasizes that ocular-specific molecular factors, such as TGF-B signaling, myofibroblast activity, and conjunctival tissue quality, are often stronger contributors to GDD failure than a patient’s overall systemic health or broader factors [36,37].

Future studies should aim to prospectively validate frailty indices in the context of glaucoma surgery while examining which frailty components most strongly predict postoperative complications. Studies can investigate whether preoperative interventions, including physical conditioning, nutritional supplementation, or comorbidity optimization, reduce complication rates. Future research should also explore how modifiable factors like systemic inflammation or medication burden interact with frailty to influence IOP outcomes and surgical recovery. Given the relevance of patient-centered outcomes in frail and geriatric populations, future prospective studies can evaluate quality-of-life metrics associated with glaucoma surgery. Ultimately, using standardized frailty assessments in multi-center registries could allow for risk stratification research and eventually the development of tailored perioperative care protocols for high-risk patients.

There are multiple limitations to this retrospective cohort study. The use of ICD-9 and ICD-10 diagnosis codes for patient selection may have inappropriately excluded patients with inaccurate diagnostic coding [38]. Further selection bias could have occurred due to charts missing an eFI calculation or the fact that this was a single-center study [39]. The study cohort is only generalizable to older adults who had at least two years of prior outpatient follow-up and blood pressure measurement, making it possible to have an eFI. Patients who did not have other outpatient care at our institution would not have a calculable eFI and could not be retained in the analysis. This is likely because they received their primary care at another institution or private practice and were referred to our institution for tertiary ophthalmic care. While we adjusted for several demographic variables, there was still potential residual confounding due to unmeasured clinical or socioeconomic factors not captured in the medical record. Additionally, the modest sample size of the frail subgroup necessitated combining pre-frail and frail categories for analyses. This grouping may have limited our statistical power to detect more granular differences in outcomes and surgical success between these specific categories. Given the wide confidence intervals and modest sample size, these models may have had insufficient statistical power to detect a significant association between frailty status and intraocular pressure-based success outcomes. There is also the possibility of a type 1 error. However, these data may suggest that future larger longitudinal studies are warranted to verify the findings. In the future, interventions to prevent complications may be more likely to benefit pre-frail individuals as numerous studies have noted that frailty can be difficult to reverse [40,41,42]. Moreover, the 1-year follow-up duration may not fully capture long-term changes in visual function or quality of life. Future prospective longitudinal studies with a longer 2–3-year follow-up may be warranted to better assess these outcomes. The IOP and visual acuity measurements may not have captured elevated or decreased values in between each clinical visit. Finally, our findings may not be generalizable to other glaucoma patient populations as this study was conducted at one regional healthcare facility. Because data were restricted to patients within our system who met the age threshold and had an available eFI, these results may not apply to younger individuals or those who receive primary care externally.

## 5. Conclusions

Pre-frailty/frailty was associated with increased vulnerability to postoperative complications but not overall successful IOP reduction. The elevated complication risk highlights the importance of frailty assessment in preoperative planning for older adults. Future studies should evaluate how integrating frailty screening into perioperative discussions may optimize outcomes and support shared decision-making in aging patients undergoing glaucoma surgery. Longitudinal analyses with larger cohorts may help clarify interactions between frailty, comorbidities, and long-term shunt outcomes.

## Figures and Tables

**Table 1:** Demographic information of study patients.

Patient Demographic Information (*n* = 200)	
**Age (years)**	
Mean (SD)	70.6 (9.6)
**Sex—*n* (%)**	
Female	90 (45.0)
Male	110 (55.0)
**Marital Status—*n* (%)**	
Divorced	23 (11.5)
Married	102 (51.0)
Single	31 (15.5)
Other	3 (1.5)
Widowed	41 (20.5)
**Race—*n* (%)**	
Asian or Pacific Islander	4 (2.0)
Black	70 (35.0)
Other	4 (2.0)
White	122 (61.0)
**Ethnicity—*n* (%)**	
Hispanic	13 (6.5)
Not Hispanic	187 (93.5)
**Smoking History—*n* (%)**	
Current	18 (9.0)
Previous	95 (47.5)
Never	87 (43.5)
**Frailty Status—*n* (%)**	
Frail	29 (14.5)
Pre-Frail	91 (45.5)
Fit	80 (40.0)

**Table 2:** Glaucoma diagnosis and surgical follow-up of study patients.

Patient Glaucoma Diagnosis and Surgical Information	
**Surgery Eye—*n* (%)**	
OD	112 (56.0)
OS	88 (44.0)
**Stage—*n* (%)**	
Indeterminate	41 (20.5)
Mild	12 (6.0)
Moderate	21 (10.5)
Severe	126 (63.0)
**Shunt—*n* (%)**	
Ahmed	112 (56.0)
Baerveldt	88 (44.0)
**Cataract Combination—*n* (%)**	
No	167 (83.5)
Yes	33 (16.5)
**Glaucoma Diagnosis—*n* (%)**	
Primary Open-Angle	103 (51.5)
Pseudoexfoliation	18 (9.0)
Neovascular	36 (18.0)
Steroid	5 (2.5)
Uveitic	19 (9.5)
Traumatic	6 (3.0)
Chronic Angle Closure	10 (5.0)
Other	3 (1.5)
**Postoperative Complications—*n* (%)**	
Anterior Chamber Hemorrhage	21 (10.5)
Persistent Choroidal Effusion/Detachment	9 (4.5)
Cystoid Macular Edema	4 (2.0)
Cyclophotocoagulation	2 (1.0)
Persistent Hypotony	17 (8.5)
Persistent Inflammation	12 (6.0)
Retinal Detachment	1 (0.5)
Tube Exposure	2 (1.0)
Extra Tube	1 (0.5)
Tube Revision	0 (0.0)
None	131 (65.5)
**Outcome at 1 Year—*n* (%)**	
Partial Success	158 (79.0)
Complete Success	103 (51.5)

Abbreviations: OD, oculus dexter; OS, oculus sinister.

**Table 3:** Univariate and multivariate Cox proportional hazards models for time to first postoperative complication.

Characteristic	Reference Group	Univariate Model			Multivariate Model ^a^		
		HR	95% CI	*p*-Value	HR	95% CI	*p*-Value
**Frailty Status**							
Pre-Frail/Frail	Fit	1.51	0.90–2.53	0.118	1.90	1.07–3.36	0.027
**Age**	-	1.02	1.00–1.05	0.095	1.03	1.00–1.07	0.073
**Sex**							
Female	Male	0.92	0.56–1.51	0.746	0.99	0.55–1.75	0.961
**Marital Status**							
Married		1.41	0.76–2.61	0.280	1.12	0.55–2.31	0.748
Divorced	Single	1.92	0.73–5.03	0.184	1.31	0.47–3.70	0.604
Widowed		1.25	0.60–2.62	0.554	0.61	0.25–1.48	0.279
**Race**							
Black		0.75	0.45–1.26	0.279	0.80	0.46–1.41	0.442
Asian or Pacific Islander	White	3.07	0.41–23.02	0.275	5.50	0.68–44.66	0.111
Other		0.59	0.08–4.36	0.608	0.63	0.07–5.31	0.668
**Baseline IOP**	-	0.61	0.17–2.06	0.433	-	-	-
**Glaucoma Subtype**							
POAG	Other	1.15	0.69–1.93	0.581	-	-	-
**Shunt**							
Ahmed	Baerveldt	1.47	0.87–2.47	0.151	-	-	-
**Cataract Combination**							
Yes	No	1.42	0.74–2.73	0.294	-	-	-

^a^ Multivariate Cox proportional hazards model adjusted for demographics (age, sex, marital status, race).

**Table 4:** Odds ratios for pre-frail/frail vs. fit status (reference group) for success outcomes.

Outcome	Univariate Model			Multivariate Model ^a^		
	OR	95% CI	*p*-Value	OR	95% CI	*p*-Value
Partial Success ^b^	1.31	0.66–2.61	0.438	1.49	0.72–3.07	0.283
Complete Success ^c^	1.11	0.63–1.95	0.729	1.03	0.57–1.86	0.927

^a^ Multivariate model adjusted for demographics (age, sex, marital status, race). ^b^ Partial success denotes subjects that achieved adequate IOP control only (IOP of 5–21 mmHg and ≥20% reduction from preoperative IOP). ^c^ Complete success denotes subjects that achieved adequate IOP control (IOP of 5–21 mmHg, ≥20% reduction from preoperative IOP) and no postoperative complications.

## Data Availability

The data presented in this study are available on request from the corresponding author due to patient privacy concerns.

## Supplementary Materials

The following supporting information can be downloaded at https://www.mdpi.com/article/10.3390/geriatrics11050114/s1: Table S1: Definitions for each postoperative complication; Table S2: 54 deficits included in the eFI calculation; Figure S1: Kaplan–Meier analysis of postoperative complication timing by frailty status.

## Abbreviations

The following abbreviations are used in this manuscript:

eFI	Electronic frailty index
IOP	Intraocular pressure
VA	Visual acuity
