# Supplementary material for: The Association of an Electronic Frailty Index with Glaucoma Shunt Outcomes
Source: Geriatrics (Basel). 2026 Aug 31;11(5):114. doi: 10.3390/geriatrics11050114 (PMC13600021; doi:10.3390/geriatrics11050114)
Supplement: Supplementary file 1 [file geriatrics-11-00114-s001.zip › geriatrics-4147125-supplementary.pdf]

Supplemental Materials

Supplemental Table S1

Complication	Definition
**Tube Revision**	Any return to the operating room for surgical modification, repositioning, shortening, or repair of the implanted tube.
**Tube Exposure**	Conjunctival erosion with exposure of the tube or plate.
**Tube Removal**	Surgical explantation of the glaucoma drainage device.
**Tube Infection**	A clinically diagnosed infection involving the glaucoma drainage device, with or without consequent surgery.
**Endophthalmitis**	A clinically diagnosed intraocular infection of ocular fluids – aqueous and vitreous - and tissues, with or without vitrectomy that have required documentation of infection and may require a tap and inject.
**Persistent Choroidal (Effusion/Detachment)**	Fluid accumulation in the suprachoroidal space persisting across consecutive clinical evaluations for ≥ 2 weeks post-surgery or requiring medical/surgical management.
**Persistent Inflammation**	Persistent anterior chamber inflammation of ≥ 2 + cells and/or flare documented more than 1 month postoperatively.
**Persistent Hypotony**	Intraocular pressure ≤ 5 mmHg recorded on two consecutive follow-up visits at least 2 weeks apart.
**Retinal Detachment**	Recorded based on the treating ophthalmologist's documented clinical diagnosis.
**Choroidal Hemorrhage**	Recorded based on the treating ophthalmologist's documented clinical diagnosis.
**Hyphema (Anterior Chamber Hemorrhage)**	Recorded based on the treating ophthalmologist's documented clinical diagnosis.
**Cystoid Macular Edema**	Recorded based on the treating ophthalmologist's documented clinical diagnosis.

Supplemental Table S1. Definitions for Each Post-Operative Complication. A table highlighting how each post-operative complication was defined and measured throughout the study.

Supplemental Table S2

Area	Characteristic	Area	Characteristic
Morbidity	Anemia	Vital Signs and Laboratory Measures	Body Mass Index
	Atrial Fibrillation		Blood Pressure
	Stroke/TIA		Kidney Function
	Kidney Disease		Glucose
	Diabetes		Cholesterol
	Fragility or Fracture		Triglycerides
	Heart Failure		Potassium
	Valvular Disease		Sodium
	Hypertension		Liver Function
	Hypotension/Syncope		Mean Corpuscular Volume
	Heart Disease		Blood Urea Nitrogen
	Osteoporosis		Calcium
	Pulmonary Disease		Albumin
	Thyroid Disease		Total Protein
	Urinary Incontinence / Disease	Cognition and Mood	Dementia / Cognitive Problems
	Liver Disease		Delirium
	Cancer		Depression
	Peptic Ulcer		Stress
	Skin Ulcer		Self-Reported General Health
Function	Arthritis	Sensory Loss	Hearing Impairment
	Falls		Visual Impairment
	Activity Limitation	Other	Weight Loss
	Peripheral Vascular Disease		Chronic Pain
	Parkinson's disease		Polypharmacy
	Dizziness or Vertigo		Smoking
	Dyspnea		

Supplemental Table S2. 54 Deficits included in the eFI calculation. A table highlighting each of 54 clinical deficits included in the eFI calculation, as described and copied from Khanna et al 2023.

Supplemental Figure S1

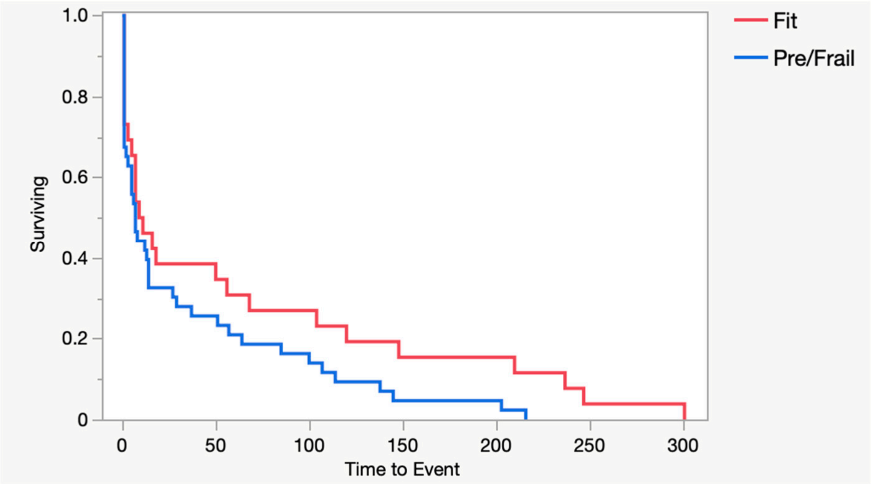

Supplemental Figure S1. Kaplan-Meier Analysis of Post-Operative Complication Timing by Frailty Status. A Kaplan-Meier survival curve demonstrating the difference in complication timing by frailty status
